# International reflections on caring for people with advanced dementia

**DOI:** 10.1111/phn.12572

**Published:** 2018-12-18

**Authors:** Yoshie Yumoto, W. George Kernohan, Noriko Morioka, Yasuko Ogata

**Affiliations:** ^1^ Graduate School of Health Care Sciences Tokyo Medical and Dental University (TMDU) Tokyo Japan; ^2^ Faculty of Life and Health Sciences Institute of Nursing and Health Research, University of Ulster Antrim UK

**Keywords:** cross‐cultural comparison, dementia, Japan, northern Ireland, palliative care

## Abstract

Dementia is causing global concern with its massive impacts on affected individuals, families, society, and national economies. As the disease progresses, patients’ needs increase in number, depth, and breadth, covering physical, psychological, social, and spiritual domains. Care varies from place to place, from country to country and from east to west. To learn from some of these variations, we explored advanced dementia care in United Kingdom and Japan. Informed by an overview of literature on care of people with advanced dementia, we reflected on direct nonparticipant observations of care in urban areas of Northern Ireland and Japan. While we identified a common purpose to address the complex needs of people living with dementia, there were differences in the approach to care. Broadly, dementia care in United Kingdom tends toward person‐centered care with a strong interest in Advance Care Planning as part of a palliative care approach. In Japan, we found less evidence of early stage palliative care and more of family‐based decision making to inform care of older people. In both countries, dementia care varies regionally, being more available in some areas than others. International knowledge exchange and further comparative studies will help to improve care for people with advanced dementia, everywhere.

## 1 INTRODUCTION

Almost 50 million people around the world are living with a dementia diagnosis and the number of new cases is increasing by about 7.7 million per year (World Health Organization, 2012). The condition affects mostly older people and has a long trajectory (Li et al., 2017; Verlinden et al., 2016). It starts off with the characteristic symptom of memory loss and ends with an inability to live daily life independently. Dementia has a huge impact on social, economic, and medical systems worldwide (World Health Organization, 2012). Many countries are attempting to address the challenge of dementia in their national strategies and action plans (Nakanishi et al., 2015; World Health Organization, 2012). Although the approaches within these national plans and strategies vary from country to country, reflecting different social structures and policy situations, the worldwide priority actions are agreed to be awareness raising and prevention; early diagnosis; commitment to good quality continuing care and services; caregiver support; workforce planning and effective training; research to better understand the disease and inform evidence‐based care (World Health Organization, 2012).

Dementia is recognized as a long‐term life‐limiting illness: as yet there is no cure or effective treatment (Koller et al., 2012; Lee & Chodosh, 2009). With such a lengthy deterioration, patients often develop comorbidities and at the same time, lose the ability to communicate (Prince, Comas‐Herrera, Knapp, Guerchet, & Karagiannidou, 2016). Pain and discomfort are common concerns, but poor communication makes it difficult to identify, locate, and treat chronic pain and other symptoms. It is important that health care professionals around the world understand the complexity of dementia, so they can better support those affected in different settings, particularly toward the end of life. In this paper, we review approaches to the care of people with advanced dementia in United Kingdom and Japan, based on appraisal of current evidence combined with nonparticipant observation in both countries.

## 2 BACKGROUND

From a global perspective, dementia is understood and addressed within many different social, economic, organizational, and cultural dimensions. The way people deal with these challenging situations and the variable support provided reflects local cultural influences. Broadly stated, individualism is predominant in Western society, while collectivism is more dominant in decision making in the East (van der Steen et al., 2013; Matsumura et al., 2002). These and many other cultural differences offer an opportunity to compare the actual situation between countries, in order to identify potential improvements in quality of care.

Japan and United Kingdom have broadly similar health care systems based on universal health coverage, with essential health services made available to those who need them, either free or at low cost. Generally, health services are controlled by central or regional government, while social care services are often provided through the local authorities. Subtle differences persist between and within each region. In Japan, the universal coverage for elders is supported through long‐term care insurance (LTCI) (Campbell & Ikegami, 2000; Matsuda & Yamamoto, 2001). A similar National Insurance scheme operates in United Kingdom, with a small proportion of care being privately funded, although arrangements vary across the U.K. nations, as the governance arrangements are devolved to local government in England, Scotland, Wales, and Northern Ireland (Doheny, 2015).

Even though the multiple differences make comparison within and between countries complex, the two countries address elder care within a similar health insurance system; in other words, care for older people is provided based on universal coverage. This broadly similar organizational approach makes it possible to focus on the cultural differences (other than funding systems) which directly affect decision making regarding care to address the primary and common concerns, that is, to maintain the quality of life of the people with dementia and their families.

People living with dementia have unique needs, because the distinctive nature and course of disease lead to physical and psychological deterioration. Professional carers must meet the complex needs of those with dementia and their families. People living with dementia need help with many aspects of daily life: professional care to provide comfort, nutrition, and symptom control; specific help with communication and decision making; care to address emotional and psychosocial needs (Lawrence, Samsi, Murray, Harari, & Banerjee, 2011; Lee et al., 2017).

The social and cultural differences between regions provide a rich opportunity to explore natural variation in the way services are provided. Thus, the aim of this study was to examine and compare the care provided to people living with advanced disease in selected settings in Japan and United Kingdom. Cognizant of global consensus upon the twin goals of (a) person‐centered care, communication, and shared decision making; and (b) optimal treatment of symptoms and providing comfort (Lawrence et al., 2011; Lee et al., 2017; van der Steen et al., 2014), we set our focus on these two aspects in our two countries.

Hence, this comparative study included an overview of relevant literature and direct nonparticipant observation in urban areas in both countries, with a focus upon physical aspects of care, communication, and decision making. Our purpose was to better understand dementia care across United Kingdom and Japan, in order to inform practice development.

## 3 METHODS

To gather existing knowledge on care of people with advanced dementia, an exploratory overview of published papers, including policy documents and reports relating to both countries was completed (September 2017). Relevant literature was identified through MEDLINE and CINAHL. The search terms used were “dementia,” “end‐of‐life,” combined with “United Kingdom” or “Japan.” Our focus was mainly on “communication and decision making”; “care for comfort” and “symptom management”; these were the search terms. The same terms were also used to access gray literature (using Google and Google Scholar) to obtain reports or guidelines issued by government, professional societies, or private research institutions. We restricted the search to the English language or Japanese. No formal restriction on publication date was applied. Titles and abstracts were screened and the articles which focused on disease other than dementia and those with unreachable full‐text sources were excluded.

The literature was supplemented with information derived from visits to clinical sites located in urban areas in both countries; nonparticipant observation of care facilities in Northern Ireland, United Kingdom and reflection upon experience in practice settings in Tokyo, Japan.

## 4 FINDINGS

Results are presented according to two main themes: (a) decision making and (b) symptom management. These are presented below, each with a United Kingdom and Japan perspectives.

### 4.1 Decision making in United Kingdom

Thorough communication with patients is mandatory in United Kingdom for consent purposes and in making decisions about individual care. However, effective communication is made difficult in cognitive decline as the disease progresses. In such cases where the patient loses the ability to make decisions, maintaining their autonomy on decision making is difficult.

In United Kingdom, there are several strategies and Acts of Parliament which address decision making for the patients whose ability to decide is lost. The 2005 Mental Capacity Act (Ministry of Justice, 2007) allows a family member to make decisions, only where they have been given lasting power of attorney. Taking the legal aspects forward, the End‐of‐Life Care Strategy (Department of Health, 2008) and the Prime Minister's Challenge (Department of Health, 2015) called for the autonomous decision making, promoting Advance Care Planning (ACP). It is intended to be an effective tool to identify patients’ needs and preferences early in the disease process in order to mitigate family conflict that often arises later, when making decisions on behalf of patients.

While over a third (36%) of the U.K. population has written a will, only 8% of people in England and Wales have completed ACP (South East Coast Clinical Senate, n.d.). Generally medical doctors take responsibility for initiation of ACP (van der Steen, Galway, Carter, & Brazil, 2016), ideally at diagnosis, or soon thereafter (Dening, King, Jones, Vickestaff, & Sampson, 2016) including for people with dementia (Poppe, Burleigh, & Banerjee, 2013).

After many years of development, the Mental Capacity Act (Northern Ireland) gained Royal Assent in May 2016 (Lynch, Taggart, & Campbell, 2017). It will encourage a less paternalistic and more person‐centered approach to decision making so that everyone's wishes are respected, as far as possible. The new law is unique in that it makes it possible for specific safeguards at the time when someone “lacks capacity” regarding a specific decision. During our visit, we heard from senior staff that the new law has yet to be fully implemented, as associated papers are needed to guide practice. It will also need a significant change in thinking and culture; a move away from a paternalistic view that the professional knows best, toward an emphasis on the choices, rights, feelings, values and beliefs of the person being cared‐for.

### 4.2 Decision making in Japan

In Japan, it is common for decisions about end‐of‐life care, especially regarding an older patient when they become frail, to be made mainly by family or sometimes by medical professionals (Ito, Tanida, & Turale, 2010; Konishi, Davis, & Aiba, 2002; Nakanishi & Honda, 2009). Only half of Japanese doctors give priority to their patients’ wishes for medical care, regardless of the patient's competency (Asai, Miura, Tanabe, Kurihara, & Fukuhara, 1998; Miyata, Shiraishi, & Kai, 2006).

In our experience, it is challenging to apply the decision‐making process, elicited through collective preference, not only for patients and their family but also medical professionals, who themselves experience difficulties. There is a widespread reluctance to engage in ACP. Hence, the Government of Japan now also promotes the participation of patients in decision making regarding end of life and requires the implementation of such decision‐making process as part of the reimbursement fee schedule of LTCI (Japanese Council of Senior Citizens Welfare Service, 2015). Several guidelines state how the decisions should be made when the patients are incapable of making their own decisions (All Japan Hospital Association, 2016; Japan Medical Association, 2007; Ministry of Health, Labour and Welfare [MHLW], 2015; The Japan Geriatrics Society, 2012): decisions should be made in the patient's best interests, in discussion with the families or relatives and medical professionals. However, recent guidelines refer to ACP as the decision‐making principle in respect of a patient's right (MHLW, 2018). Although substitute decision‐makers such as families and medical professionals have the ultimate responsibility on the final decision, there are no regulations to validate such decisions.

### 4.3 Symptom management in United Kingdom

The palliative approach is increasing in importance in United Kingdom to benefit those diagnosed with chronic illness through person‐centered, comfort care as well as continuity and optimal symptom control (National Collaborating Centre for Mental Health (UK), 2007). However, even with a high level of public interest and debate in United Kingdom, people with dementia tend to experience suboptimal symptom management, in spite of them enduring a similar burden to other life‐limited illness such as cancer (Davies et al., 2014; Sampson, 2010).

Initially U.K. hospices began with a focus upon cancer, but are now expanding the palliative approach to other nonmalignant diseases including dementia. At the same time, they are moving to provide palliative care in various care settings, including at home and at outpatient clinics known as “Day Care.” For example, we visited one hospice in Northern Ireland that runs a special palliative care program for people with dementia. In special dementia‐friendly facilities, they provide services specialized for dementia, with interior design used effectively for less confusion, employing staff who are skilled and specialized in dementia care. Nevertheless, care for the person with dementia can be challenging due to wide variation in context, with practical complexities in addressing a wide range of symptoms that have been described as “chaotic” with highly variable needs that require a flexible approach (Davies et al., 2014) with interdisciplinary collaborations (Ryan, Gardiner, Bellamy, Gott, & Ingleton, 2012) across a wide range of settings: those in remote rural settings can be particularly disadvantaged.

In United Kingdom, the majority of people with a cause of death recorded as dementia die in hospital, care home, nursing home, or in the community, with very few (0.3%) in a hospice (Sleeman, Ho, Verne, Gao, & Higginson, 2014). “The gold Standards Framework” (Hansford & Meehan, 2007), which offers educational modules about palliative care, provides guidance for optimal care in those settings and several evidence‐based interventions or protocols are under development in England (Davies, Manthorpe, Sampson, & Iliffe, 2015; Jones et al., 2012).

### 4.4 Symptom management in Japan

To our knowledge, symptom management in advanced stages of dementia has not been actively discussed in Japan, such that symptoms at end of life might be more appropriately managed. In our experience, residents in Japanese nursing homes tend to undergo somewhat burdensome interventions such as tube feeding, cardiopulmonary resuscitation, and hospital transfer at the end of life (Nakanishi & Miyamoto, 2016).

A private sector survey reported that over 60% of long‐term care facilities provide end‐of‐life care for older people (Mizuho Information & Research Institute, Inc., 2014). Staff are often highly motivated to provide good end‐of‐life care and establish systems and policies to ensure quality care, such as care manuals with training programs for all staff. End‐of‐life care is not specific to dementia rather the focus of end‐of‐life care in long‐term care facilities is on the patient's quality of life with appropriate symptoms control, which is called *Mitori care* (Japanese Council of Senior Citizens Welfare Service, 2015). Mitori care is similar to Palliative care, but its application is limited to the terminal phase (Japanese Council of Senior Citizens Welfare Service, 2015). Quality of care at the end of life in those facilities has recently been promoted by a financial bonus system which regulates the eligibility criteria on the facilities and patients to receive *Mitori care*. Payment starts 30 days before death and is claimed retrospectively (Nishiguchi, Sugaya, Sakamaki, & Mizushima, 2017). This reflects the fact that more resources are required to meet the needs of people with terminal illness.

Few articles regarding palliative care focusing on people with dementia in Japan were found in our research. One of them reports the implementation of palliative care to people with chronic disease, including dementia, in one hospice (Nishikawa et al., 2013); the other was a large‐scale cross‐sectional study which examines the correlation between staff knowledge about palliative care and their attitudes (Nakanishi & Miyamoto, 2016). Implementing palliative care for people living with nonmalignant disease is not yet common in Japan; under the public health insurance scheme, most hospice services are reserved for patients with advanced cancer (Nakanishi & Miyamoto, 2016).

## 5 DISCUSSION

We reviewed approaches to the end‐of‐life care of people with advanced dementia in United Kingdom and Japan, based on literature, other relevant documents and nonparticipant observations, in both countries. We focused on the critical elements of end‐of‐life care for advanced dementia: *Communication & decision making* and *Care to provide comfort*. Although these elements are only part of end‐of‐life care, we used them to focus attention upon quality care of patients with dementia in both countries. There were differences in the approaches of both countries: some approaches to care in United Kingdom are highly specialized for dementia, led by government strategies or local agencies. Although our study reviewed practices in a limited way, we find that Government Acts and regional policies help to guide services and promote good quality end‐of‐life care for people with dementia. However, not many people benefit from such specialized services for dementia. In Japan, we found a highly systemized provision of care for older people who need support in all care settings, under the LTCI scheme that covers medical and welfare services including end‐of‐life care both in community and inpatient facilities (Campbell & Ikegami, 2000). Care for people with dementia in Japan is provided within the existing services for older people with any diagnosis: though services are organized within a good infrastructure, they are not developed specifically for people with dementia.

### 5.1 Communication and shared decision making

Many U.K. health policies encourage the identification of patients’ individual needs and preferences. ACP is promoted as a way to achieve autonomy, in advance of cognitive impairment. It can be an effective tool even for people diagnosed with dementia. Some elements such as the timing need to be examined to optimize its use. In many situations, family members are left to take substitute decisions, on behalf of people who have lost capacity (Dening et al., 2016; Livingston et al., 2010).

It is true that Japanese families and care professionals tend to make decisions on behalf of elders (Kuraoka & Nakayama, 2014). However, recognition of the importance of involving patients themselves and their families together in decision‐making processes has been increasing (Miyata et al., 2006). These processes are culturally rooted, so it is necessary to ensure a culturally “fit” and achieve family agreements that honor patient preference and lead to optimal care for them.

Both United Kingdom and Japan struggle with end‐of‐life decision making, respecting the patient's own free choice, while avoiding family conflict. However, an effective system to support decision making is a “must” for good quality end‐of‐life care. Further research is needed to examine the effectiveness of timely ACP to promote decision making while ensuring good cultural fit.

### 5.2 Optimal treatment of symptoms and providing comfort care

Globally, high quality palliative care can overcome burdensome and unique physical, psychological, social, and spiritual needs in different ways, in various styles. The provision of dementia care in Northern Ireland was remarkable because it can provide disease‐specific; dementia‐friendly care until the end of life. The services we saw cover all the essentials for people with dementia (Prince et al., 2016): continuous, holistic, and integrated health care. Here, patients receive palliation earlier in disease trajectory than in most other models of care. In Japan, patients can also access continuous and standardized care. However, the care is not disease‐specific, tends toward *Mitori care*, and is less focused on symptom management or palliation. There is a need to develop optimal palliative care for people in earlier stages of dementia to achieve quality of life throughout the disease trajectory, even from diagnosis.

Indeed, all this assumes that dementia is correctly diagnosed in a timely fashion: a critical first step in addressing specific needs (Nakanishi et al., 2015). However, the diagnosis rate is still low, even in high income countries (Prince et al., 2016). By improving diagnosis, engaging with ACP and thereafter addressing symptom control, it may be possible to achieve good quality care for all.

### 5.3 Limitations and recommendations

We explored the situation at end of life for people with dementia in United Kingdom and Japan through literature overview and direct observations in Northern Ireland and Tokyo. The challenges of care are similar in both settings: achievement of effective symptom control within a context of clear communication. Recognizing the limitations of observational study, which cannot provide evidence of clinical effectiveness, we provide some useful reflections to build a foundation for further comparison and learning. We found many similar situations between the countries: both striving to provide optimal care for end‐of‐life care in dementia, albeit using different approaches: U.K. services being more person‐centered and government driven, while in Japan, dementia services depend more on the culture of family‐centered support, each with unique local care systems and each influenced by the dominant funding model in the region. Developments in Health Insurance are influential in Japan and the recent inclusion of ACP will promote its introduction in palliative care, which for the moment, still remains focused largely on people with cancer. ACP in dementia is complex and requires sensitive application, family engagement and evaluation. To advance the quality of care for people with dementia, professionals in public health have an important role: early in the disease process, people with dementia need support, information, and access to care near their home. In any setting, access to specialized care is limited, so a better understanding by professionals, carers, and people with dementia is likely to enhance quality of life. We need to help people with dementia and their family to prepare for their upcoming experience and make decisions in advance of cognitive decline (via ACP).

Ongoing knowledge exchange is needed in order to address the needs of people with dementia and their families, across all four palliative care domains of physical, psychological, social, and spiritual care (World Health Organization, n.d.). Further research work is needed to underpin this knowledge exchange in order to compare and contrast different components of care. It is important to recognize the value of international comparison and collaboration in this area.

## ACKNOWLEDGEMENT

The authors thank participating staff for providing information and facilitating site visits in Northern Ireland. This work was supported by JSPS KAKENHI Grant Number JP16K20814‐00.

## CONFLICT OF INTEREST

The authors declare no conflicts of interest.
